# Combination of mitomycin C and low-dose metronidazole synergistically against *Clostridioides difficile* infection and recurrence prevention

**DOI:** 10.1128/aac.00515-25

**Published:** 2025-06-17

**Authors:** Jun-Jia Gong, I-Hsiu Huang, Yuan‑Pin Hung, Yi-Wei Chen, Yun-Chien Lin, Jenn-Wei Chen

**Affiliations:** 1 Institute of Basic Medical Sciences, College of Medicine, National Cheng Kung University 593433, Tainan, Taiwan; 2 Department of Biochemistry and Microbiology, Oklahoma State University Center for Health Sciences 33264, Tulsa, Oklahoma, USA; 3 Department of Microbiology and Immunology, College of Medicine, National Cheng Kung University 38026, Tainan, Taiwan; 4 Department of Internal Medicine, College of Medicine, National Cheng Kung University Hospital, National Cheng Kung University 38026, Tainan, Taiwan; 5 Department of Medicine, College of Medicine, National Cheng Kung University 38026, Tainan, Taiwan; 6 Division of Oral Biology and Medicine, School of Dentistry, University of California 49038, Los Angeles, California, USA; The Peter Doherty Institute for Infection and Immunity, Melbourne, Victoria, Australia

**Keywords:** *Clostridioides difficile*, mitomycin C, metronidazole, spore, recurrence

## Abstract

*Clostridioides difficile* is an anaerobic, spore-forming pathogen responsible for illnesses ranging from mild diarrhea to life-threatening colitis. Current treatments rely on antibiotics such as vancomycin and metronidazole (MTZ), but high doses can disrupt gut microbiota, contributing to recurrent infections. Mitomycin C (MMC), a Food and Drug Administration-approved anticancer agent, is known to induce prophage activation in lysogenic bacteria. Given that over 70% of *C. difficile* strains harbor prophages, we evaluated MMC’s potential to enhance antibiotic efficacy against *C. difficile* infection (CDI). *In vitro*, MMC synergized with MTZ to inhibit strain R20291 and clinical isolates of RT027 and RT078 while reducing the minimum bactericidal concentration of MTZ against biofilm-associated cells. Ex vivo assays using mouse fecal suspensions confirmed the enhanced killing effect of the combination. In a murine recurrence model, low-dose MTZ + MMC treatment significantly improved survival and reduced fecal spore counts compared to monotherapies or vancomycin. Importantly, the combination did not cause greater liver or kidney toxicity than other antibiotics and resulted in less colonic epithelial damage. Microbiota profiling revealed that MTZ + MMC better preserved gut microbial composition than standard treatments. These findings suggest that low-dose MTZ combined with MMC enhances antimicrobial efficacy while reducing toxicity and microbiota disruption, offering a promising strategy for CDI management.

## INTRODUCTION

*Clostridioides difficile* is an anaerobic, gram-positive, spore-forming bacterium. The symptoms of *C. difficile* infection (CDI) can range from asymptomatic carriage and mild diarrhea to severe pseudomembranous colitis and even death (1). Antibiotics are clinically recommended against CDI (2–4). However, high doses of antibiotics can disrupt the host gut microbiota, leading to recurrent *C. difficile* infection (rCDI) (5). rCDI is a serious clinical challenge, with over 20% of patients experiencing recurrence within 30 days (6), and 60% of those will relapse again (7). Notably, this high recurrence rate (60%) is generally observed in patients who have already experienced multiple episodes of CDI (8). The mortality rate for primary CDI is 2.7%, while for rCDI, it is 25.4%, which is nearly 10 times higher (9). Therefore, investigating effective ways to reduce rCDI is crucial.

Repurposing Food and Drug Administration (FDA)-approved non-antibiotic drugs for antimicrobial therapy has recently gained significant traction as a discovery method (10–12). Mitomycin C (MMC), an FDA-approved anticancer drug, inhibits DNA synthesis by cross-linking DNA (13). In addition to treating several cancer diseases (14–16), MMC also exhibits antibacterial activity (17, 18) and exhibits potent activity against *C. difficile* (19). Combining an antibiotic with the antineoplastic agent MMC has been suggested as a new therapeutic strategy for treating difficult-to-treat infections. For example, combining MMC with gentamicin synergistically inhibits most *Pseudomonas aeruginosa* strains *in vitro* and *in vivo*. Moreover, the combination of MMC with a tobramycin-ciprofloxacin antibiotic hybrid has been shown to be effective against various multidrug-resistant gram-negative bacteria (20).

Based on these findings, we hypothesized that MMC could enhance the efficacy of antibiotics against CDI. In this study, we evaluated the potential of MMC as an adjunctive treatment for CDI. Our study demonstrated that MMC and metronidazole (MTZ) synergistically inhibited *C. difficile* RT027 and RT078 strains, reducing the minimum bactericidal concentration (MBC) of MTZ for R20291 biofilm. Additionally, the rCDI animal model trial indicated that combining low-dose MTZ with MMC effectively improved mice survival rates and reduced the spore counts in their stool.

## MATERIALS AND METHODS

### Bacterial strains and culture conditions

The bacterial strains used in this study are listed in Table S1. All *C. difficile* strains were cultured anaerobically (10% H_2_, 10% CO_2_, and 80% N_2_) at 37°C in brain heart infusion (BHI) media (Thermo Fisher Scientific, Waltham, MA, USA), supplemented as needed with 0.05% L-cysteine (Sigma-Aldrich, St. Louis, USA) and 0.5% yeast extract (Thermo Fisher Scientific). The anaerobic bacteria were maintained in an anaerobic workstation (DG250; Don Whitley Scientific Ltd., West Yorkshire, UK).

### Checkerboard assay

To determine the effects of MMC (Cyrusbioscience, New Taipei City, Taiwan) with MTZ or vancomycin (VAN) (Cyrusbioscience), we conducted the checkerboard assay in 96-well plates (SPL life sciences, Pocheon, South Korea) (21). The initial drug concentrations were prepared at 4× the minimum inhibitory concentration (MIC), followed by twofold serial dilutions. Each well was inoculated with 100 µL of *C. difficile* at 10^6^ colony-forming units per milliliter in brain heart infusion supplemented (BHIS) broth. After 18 hours of incubation at 37°C, the OD_600_ values were measured using the iMark Microplate Absorbance Reader (Bio-Rad, Hercules, CA, USA). The combined drug effects were quantified by the fractional inhibitory concentration (FIC) index as follows:


$$\text{FIC index}=\frac{\left(\text{MIC of drug A in combination}\right)}{\left(\text{MIC of drug A alone}\right)}+\frac{\left(\text{MIC of drug B in combination}\right)}{\left(\text{MIC of drug B alone}\right)},$$

where synergy is indicated by an FIC index of ≤0.5; additive or indifference is indicated by an FIC index of >0.5 but <4; and antagonism is indicated by an FIC index of >4 (22). The results represented three independent experiments with the average from triplicate measurements.

### Epsilometer test assay

The susceptibility of *C. difficile* to MTZ was determined using the epsilometer (E-test) assay (23). Overnight cultures of R20291 were diluted 1:100 in BHIS broth and grown until OD_600_ reached 1. The refreshed cultures were then spread on BHIS agar with or without MMC using sterile glass plate spreaders. An MTZ E-test strip (bioMérieux, Marcy-l'Étoile, France) was placed at the center of each plate, followed by incubation at 37°C for 24 hours. The inhibitory concentration was determined at the intersection of the inhibition zone and the test strip. This experiment was representative of three independent replicates.

### Biofilm formation and MBC assay

The biofilm formation assay was conducted as previously described (16). Briefly, overnight cultures of strain R20291 were diluted 1:100 in BHI broth supplemented with 0.1 M glucose (Avantor, Radnor Township, PA, USA) and grown until the OD_600_ reached 0.1. A total of 200 µL of cultures was added to each well of a 96-well plate and incubated anaerobically at 37°C for 72 hours. The supernatants were then replaced with 200 µL of BHI-glucose broth containing antibiotics, followed by an additional 48 hour incubation. Subsequently, the supernatants were removed, and the biofilms were resuspended in 200 µL of BHIS broth. The MBC assay was conducted to evaluate biofilm disruption (24). Two microliters of resuspended biofilm solution was plated on BHIS agar and incubated at 37°C for 24 hours. Experiments were conducted in triplicate and repeated independently three times.

### Ex vivo bacterial-fecal co-culture assay

Fresh feces from C57BL/6 mice were suspended in sterile ddH_2_O at a ratio of 0.01 g/mL. Overnight cultures of R20291 were diluted 1:100 in BHIS broth and grown until the OD_600_ reached 1. Each group received 5 × 10^5^ CFU of R20291 in 800 µL BHIS broth, with or without the following treatments: 1 µg/mL of MMC, 0.375 µg/mL of MTZ, 3 µg/mL of MTZ, or 1 µg/mL of MMC + 0.375 µg/mL of MTZ. These were mixed with 200 µL of fecal suspensions and incubated anaerobically for 24 hours. Mixtures were then diluted 10^6^-fold and spread on selective cycloserine-cefoxitin fructose agar plates for CFU quantification. Four independent biological replicates were performed, each with technical duplicates. Statistical analysis was performed using one-way analysis of variance (ANOVA) with GraphPad Prism version 10.0 (GraphPad, La Jolla, CA, USA).

### Spore purification

The spore purification process was performed as previously described (25). Briefly, overnight cultures of *C. difficile* were diluted 1:100 in BHIS broth and grown until the OD_600_ reached 0.8. Cultures were inoculated on sporulation medium for *Clostridioides difficile* (SMC) agar in six-well plates (SPL Life Sciences) and incubated anaerobically at 37°C for 7 days (26). Spores were harvested using ice-cold sterile ddH_2_O and stored at 4°C overnight. Samples were centrifuged at 2,330 × *g* for 16 min at 4°C and washed five times with ice-cold sterile ddH_2_O. Spores were then purified using Nycodenz (48%; Axis Shield, Oslo, Norway), centrifuged at 2,500 × *g* for 10 min, and washed five more times with ice-cold sterile ddH_2_O before resuspension in 1× phosphate-buffered saline (PBS) and stored at 4°C. Spores were serially diluted and plated on BHIS agar with 0.1% taurocholic acid (Sigma-Aldrich) to quantify CFUs.

### Experimental rCDI animal model and sample collection

Seven-week-old male C57BL/6 mice were obtained from the Laboratory Animal Center of National Cheng Kung University (NCKU). The rCDI model was established as described (27). Mice were administered 10 mg/kg clindamycin intraperitoneally 24 hours prior to infection, followed by oral challenge with 100 µL containing 10^4^ CFU R20291 spores. Antibiotic treatments were administered orally on days 2 and 3 post-infection. To assess the effect of MMC and MTZ co-treatment, mice were divided into six groups as described: (i) sterile 1× PBS (mock group, three mice, and R20291 group, eight mice); (ii) 50 mg/kg of MTZ dissolved in 1× PBS (MTZ_HC_ group, eight mice); (iii) 7 mg/kg of MTZ dissolved in 1× PBS (MTZ_LC_ group, eight mice); (iv) 5 mg/kg of MMC dissolved in 1× PBS (MMC group, eight mice); and (v) 7 mg/kg of MTZ combined with 5 mg/kg MMC dissolved in 1× PBS (MTZ_LC_ + MMC group, eight mice). In a separate experiment comparing MTZ_LC_ + MMC to VAN (20 mg/kg), three groups were used (*n* = 9 per group) and respectively treated with (i) 50 mg/kg of MTZ dissolved in 1× PBS (MTZ_HC_ group), (ii) 20 mg/kg of VAN dissolved in 1× PBS (VAN group), and (iii) 7 mg/kg of MTZ combined with 5 mg/kg MMC dissolved in 1× PBS (MTZ_LC_ + MMC group). Mice were monitored daily for clinical sickness scores (28), and fecal samples were collected on days 3, 5, and 7 post-infection for spore quantification. Serum was analyzed for glutamic pyruvic transaminase (GPT), glutamic oxaloacetic transaminase (GOT), blood urea nitrogen (BUN), and creatinine (CRE) using a dry biochemistry analyzer (NX-600; Fujifilm, Minato, Tokyo, Japan). Statistical analyses were conducted using GraphPad Prism version 10.0 with *t*-test and one-way ANOVA.

### Antibiotic-treated animal model and histopathological examination

C57BL/6 mice were orally administrated with PBS only, low-dose MTZ (7 mg/kg) + MMC (5 mg/kg), high-dose MTZ (50 mg/kg), or VAN (20 mg/kg) twice. After 2 days, fecal samples and colonic tissues were collected. Colons were fixed in 10% formaldehyde (BS Chemical, Lithuania), embedded in paraffin, sectioned, stained with hematoxylin and eosin, and analyzed microscopically. Inflammatory severity was assessed using criteria including neutrophil infiltration, vascular congestion, and epithelial damage (29).

### Quantification of *C. difficile* spore-forming CUFs

Fresh fecal samples from days 3, 5, and 7 post-infection were weighed and homogenized in 1 mL ddH_2_O containing 100 mg of 0.1 mm zirconia beads (BioSpec Products, Inc., Bartlesville, OK, USA) using a BeadBeater (Bertin Technologies, Avenue Ampère, Montigny-le-Bretonneux, France) for 50 s (27). Samples were heat-treated at 65°C for 20 min, plated on BHIS agar with 0.1% taurocholic acid (TCA), and incubated anaerobically at 37°C for 24 hours. CFUs were normalized to fecal weight. Three independent experiments were performed, each with technical duplicate. Statistical analysis was performed using GraphPad Prism version 10.0, with significance determined via *t*-test and one-way ANOVA.

## RESULTS

### Mitomycin C showed synergistic antibacterial activity in combination with metronidazole against *C. difficile* RT027 and RT078 clinical strains

**Fig 1:**
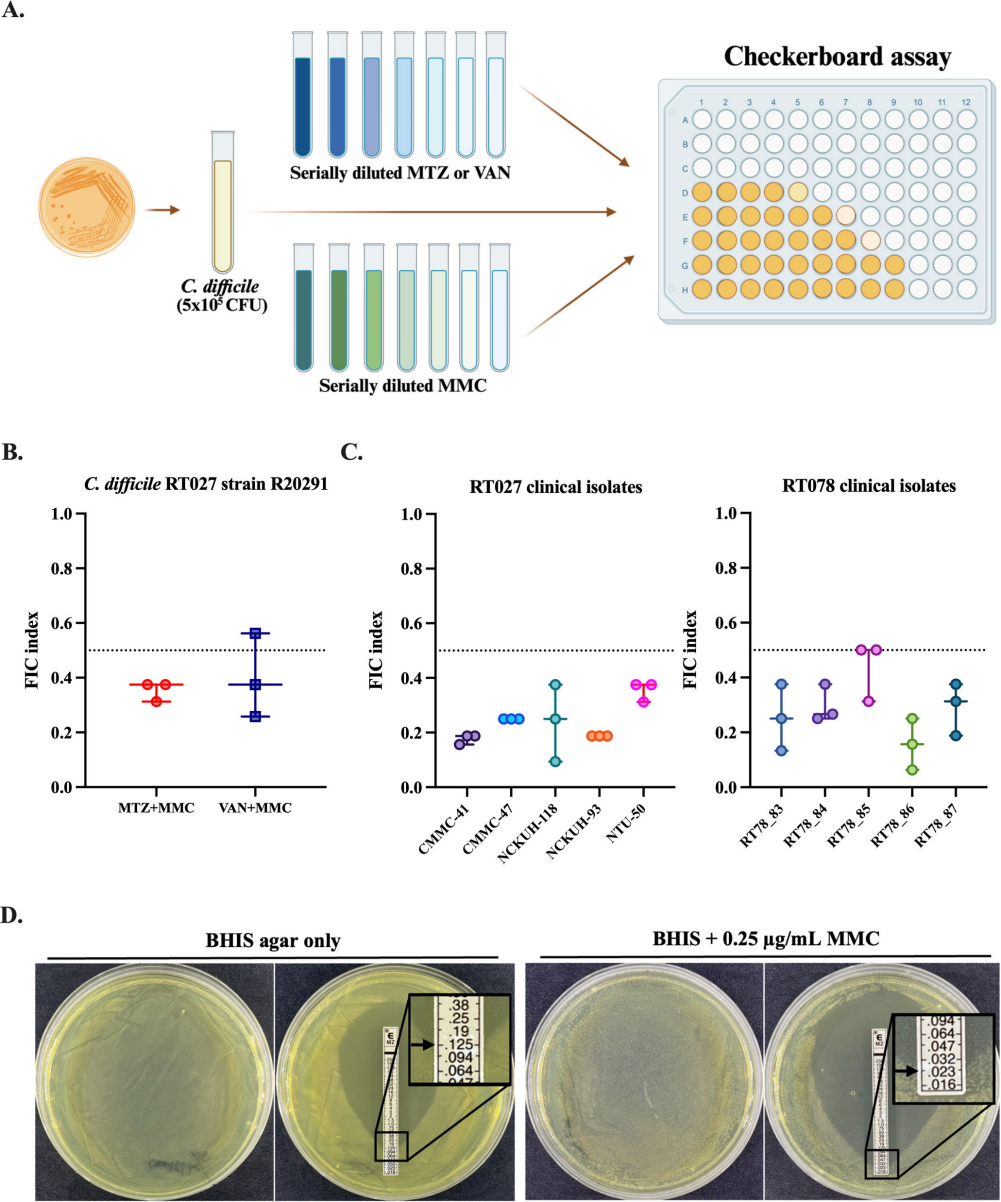
MMC and MTZ demonstrated synergistic inhibition against specific *C. difficile* strains, including RT027 (e.g., R20291) and RT078 *in vitro*. (**A**) Flowchart of checkerboard assay (created with BioRender.com). FIC index of MMC combined with MTZ or VAN when co-cultured with (**B**) *C. difficile* strain R20291 and (**C**) RT027 and RT078 clinical strains. (**D**) The MIC of MTZ alone or in combination with MMC against R20291 was determined using E-test strips. FIC, fractional inhibitory concentration; MIC, minimum inhibitory concentration; MMC, mitomycin C; MTZ, metronidazole; VAN, vancomycin. Data represent the mean of three independent experiments performed in triplicate.

A previous study used MMC to induce phage production in *C. difficile* and promote the lytic cycle (25). Interestingly, over 70% of *C. difficile* strains naturally carry prophages (30). Therefore, we investigated whether MMC could be used in combination with VAN or MTZ as a novel therapeutic strategy against CDI. We first performed a checkerboard assay (Fig. 1A), and the MIC of MMC and MTZ was determined to be 2 and 3 µg/mL, respectively, for strain R20291 (Fig. S1A). The combination of MTZ and MMC showed synergistic antibacterial activity against R20291, with an FIC index less than 0.5 (Fig. 1B). MTZ combined with 1 µg/mL MMC reduced the MIC of MTZ from 3 µg/mL to 0.375 µg/mL, an eightfold reduction (Fig. S1A). In contrast, the combination of VAN and MMC exhibited only additive activity (Fig. 1B). Furthermore, the MTZ-MMC treatment also exhibited synergistic effects against clinical isolates of *C. difficile* RT027 (CMMC-41, CMMC-47, NCKUH-118, NCKUH-93, and NTU-50) and RT078 (RT078_83, RT078_84, RT078_85, RT078_86, and RT078_87) (Fig. 1C); MMC additively enhanced VAN in inhibiting RT078 strains (Fig. S1B). These results demonstrate that MMC enhances the effects of antibiotics used to treat CDI.

E-test strips were also used to confirm the synergistic effects of MTZ and MMC against R20291. The MIC of MTZ alone was 0.125 µg/mL (Fig. 1D, left). Notably, 0.25 µg/mL of MMC alone did not inhibit bacterial growth (Fig. 1D, middle), but in combination, it reduced the MIC of MTZ to 0.023 µg/mL, a 5.4-fold reduction (Fig. 1D, right). These *in vitro* findings indicate that MTZ and MMC together are more effective in inhibiting *C. difficile* R20291 than MTZ alone.

### Mitomycin C could effectively enhance the metronidazole effect on the strain R20291 biofilms

**Fig 2:**
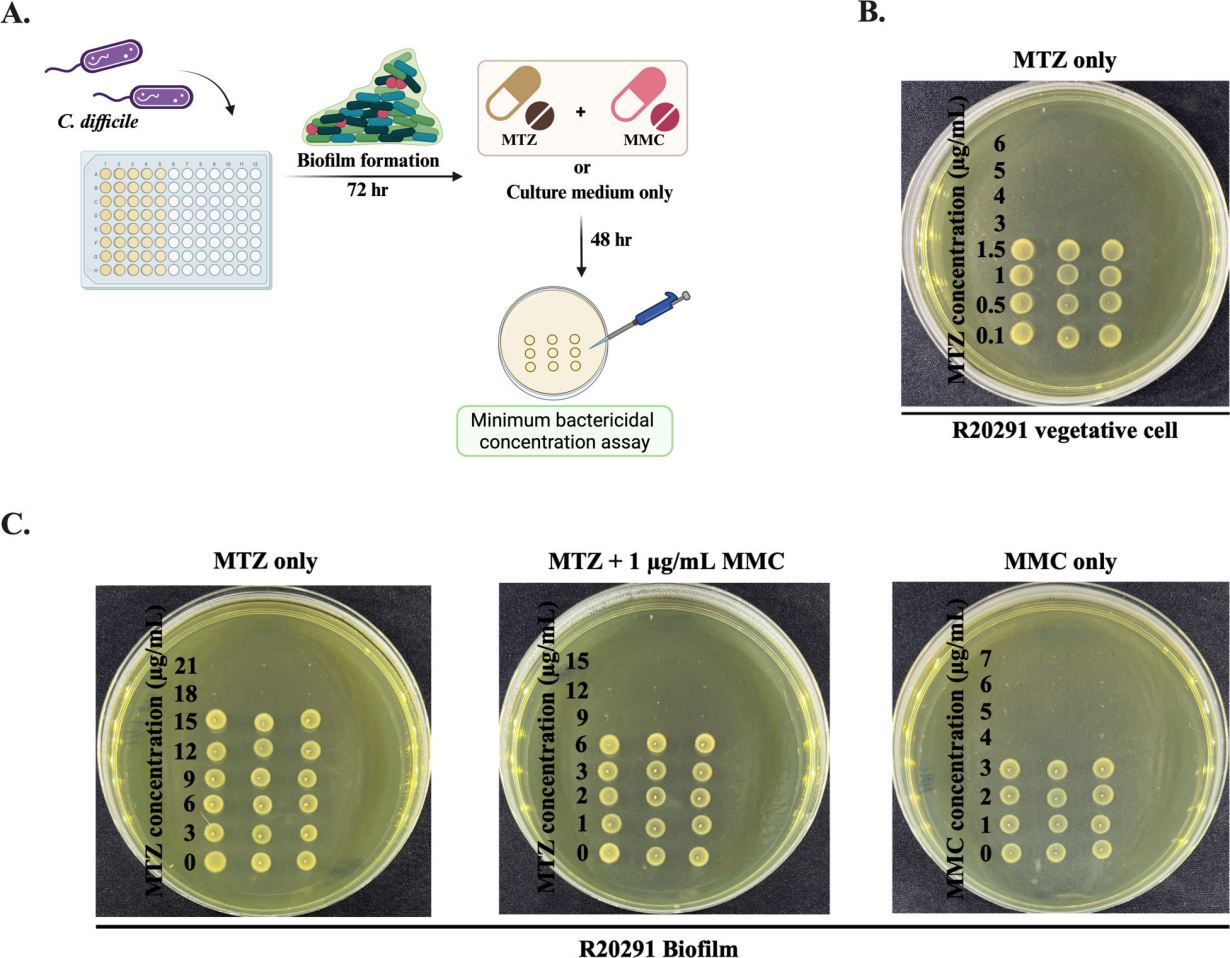
Combining MMC and MTZ effectively reduced the antibiotic resistance of R20291 biofilms. (**A**) Flowchart of antibiotic treatment protocol for R20291 biofilm formation (created with BioRender.com). (**B**) MBC assay showing the concentration of MTZ required to kill R20291 vegetative cells. (**C**) MBC values of MTZ alone, MTZ combined with 1 µg/mL MMC, and MMC alone for disrupting biofilm-associated R20291 cells. MBC, minimum bactericidal concentration. Data shown are representative of three independent experiments performed in triplicate.

In a CDI mouse model, *C. difficile* strain R20291 is known to form glycan-rich biofilms associated with increased antibiotic resistance and recurrence (31–33). Therefore, we examined whether MMC could help reduce the MTZ concentration required to eliminate biofilm-resident cells using an MBC assay (Fig. 2A). As shown in Fig. 2B, the MBC of MTZ for R20291 vegetative cells was 3 µg/mL. However, the MBC of MTZ needed to eliminate R20291 within biofilm structures increased to 18 µg/mL (Fig. 2C, left). Interestingly, combining MTZ with 1 µg/mL of MMC reduced the MBC of MTZ to 9 µg/mL (Fig. 2C, middle). MMC alone showed no toxicity to vegetative cells until concentrations reached 4 µg/mL (Fig. 2C, right). To determine if vegetative cells were released from the biofilm, we plated the biofilm supernatant on BHIS agar plates. No viable bacteria were detected outside the biofilm following antibiotic treatment (Fig. S2). Taken together, these results suggest that MMC enhances MTZ activity in targeting *C. difficile* biofilms.

### Combination of mitomycin C with metronidazole inhibits *C. difficile* colonization in fresh mouse feces

**Fig 3:**
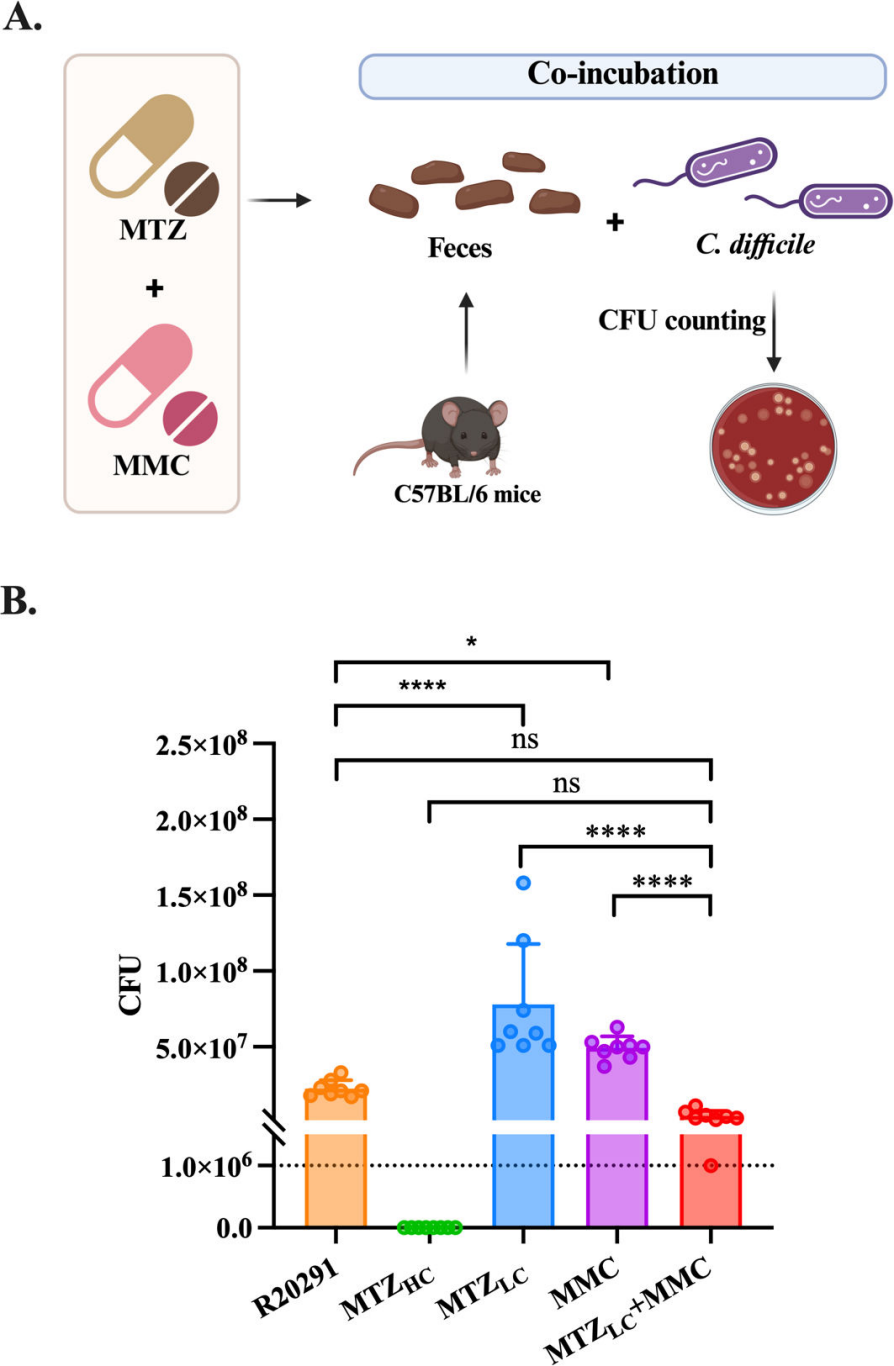
The combination of MMC and low-dose MTZ significantly inhibited the growth of R20291 in a mouse fecal environment. (**A**) Ex vivo model of R20291 co-cultured with mouse feces and treated with various antibiotic regimens (created with BioRender.com). (**B**) Fecal-bacterial mixtures were cultured for 24 hours, diluted 10^6^-fold, and plated on cycloserine-cefoxitin fructose agar to determine *C. difficile* CFU counts. MTZ_HC_, high concentration of MTZ (3 µg/mL); MTZ_LC_, low concentration of MTZ (0.375 µg/mL); MMC, 1 µg/mL MMC; MTZ_LC_ + MMC, 0.375 µg/mL MTZ combined with 1 µg/mL MMC. Data represent four independent experiments with duplicate measurements. Statistical significance was determined by one-way ANOVA. *P ≤ 0.05, ****P ≤ 0.0001. ns, not significant.

To simulate the diverse microbial gut environment, R20291 was co-cultured with fresh mouse fecal suspensions to evaluate the effect of low-concentration MTZ and MMC ex vivo. After 24 hours of incubation, 10^6^-fold diluted samples were plated to quantify CFUs (Fig. 3A). As shown in Fig. 3B, no CFU was observed in the 3 µg/mL MTZ-treated group, indicating that *C. difficile* was below 10⁶ CFU/mL. The combination of 0.375 µg/mL MTZ and 1 µg/mL MMC yielded an average of 4.5 × 10⁶ CFU/mL. In contrast, MTZ_LC_ or MMC alone resulted in 7.8 × 10⁷ and 4.9 × 10⁷ CFU/mL, respectively, both significantly higher than the MTZ_LC_ + MMC group. Interestingly, the R20291-only group had 2.25 × 10⁷ CFU/mL, which was not significantly different from the MTZ_LC_ + MMC group but remained significantly lower than the MTZ_LC_ or MMC-alone group. This may reflect the indirect effects of low-concentration antibiotics on microbial competition. These results indicate that MTZ and MMC together can suppress *C. difficile* colonization in a fecal environment.

### Treatment with metronidazole and mitomycin C increased survival and reduced spore burden in a recurrent CDI mouse model

**Fig 4:**
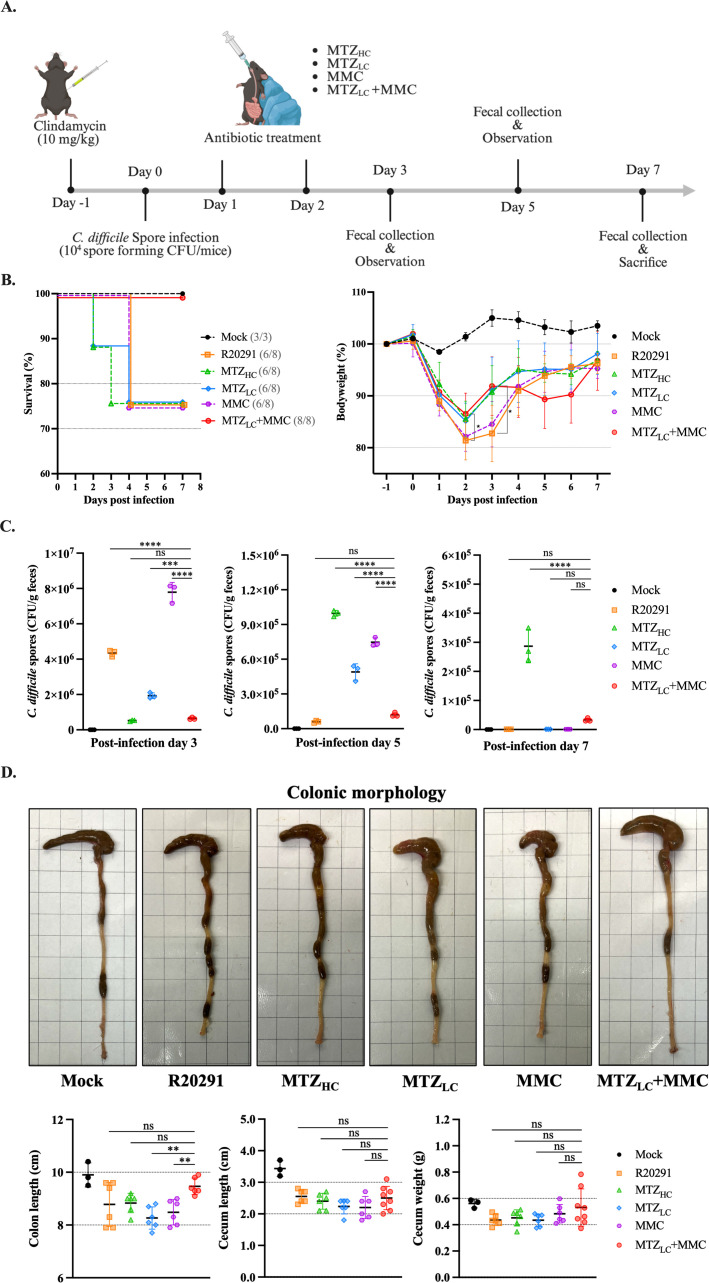
Application of low-dose MTZ combined with MMC in a mouse model of recurrent CDI. (**A**) Schematic of the recurrent CDI model in C57BL/6 mice (created with BioRender.com). Mice were pre-treated with clindamycin (10 mg/kg) and orally challenged with 10^4^ R20291 spores. Antibiotic treatments were administered on days 2 and 3 post-infection (*n* = 8 per group; mock group, *n* = 3). (**B**) Daily survival and body weight changes were recorded. (**C**) Fecal samples collected on days 3, 5, and 7 were used to quantify *C. difficile* spore CFUs. (**D**) Cecal and colonic morphology and quantification at day 7 post-infection. Each data point represents an individual mouse. Statistical analysis was performed using one-way ANOVA in GraphPad Prism version 10.0. **P ≤ 0.01, ***P ≤ 0.001, ****P ≤ 0.0001. ns, not significant.

Given the promising *in vitro* and ex vivo results, we next evaluated the therapeutic potential of MTZ and MMC *in vivo* using an rCDI mouse model (Fig. 4; Fig. S3A and B) (27). Only the non-infected and MTZ_LC_ + MMC-treated groups exhibited 100% survival, while survival in other infected groups fell below 80% (Fig. 4B, left). On day 2 post-infection, the R20291 and MMC groups exhibited the greatest weight loss, but all surviving mice recovered >95% of their initial weight by day 7 (Fig. 4B, right).

To evaluate recurrence, fecal samples were collected on days 3, 5, and 7 and plated on BHIS agar with 0.1% TCA. On day 3 post-infection, spore CFU counts were not significantly different between the MTZ_HC_ and MTZ_LC_ + MMC groups (Fig. 4C, left). However, all other infected groups had significantly higher CFU counts than the MTZ_LC_ + MMC group. On day 5 post-infection, the R20291 and MTZ_LC_ + MMC groups had significantly lower CFUs than all others, while the MTZ_HC_ group had the highest CFU burden (Fig. 4C, middle). By day 7 post-infection, all groups exhibited reduced CFUs; however, the MTZ_HC_ group still had significantly higher spore counts than the MTZ_LC_ + MMC group (Fig. 4C, right). The clinical sickness score of the MTZ_LC_ + MMC group remained lower throughout the infection period (Fig. S3C). The colonic morphology of infected mice was an important indicator for assessing intestinal inflammation (34). Histological analysis of the colon at day 7 revealed no major differences in cecum length or weight across groups, though colon length in the MTZ_LC_ + MMC group was significantly greater than that in the MTZ_LC_ and MMC-alone groups (Fig. 4D). These data demonstrate that MTZ_LC_ + MMC improves survival and reduces CDI relapse *in vivo*.

### Synergistic effects of mitomycin C and metronidazole outperform vancomycin in reducing recurrence and improving survival

**Fig 5:**
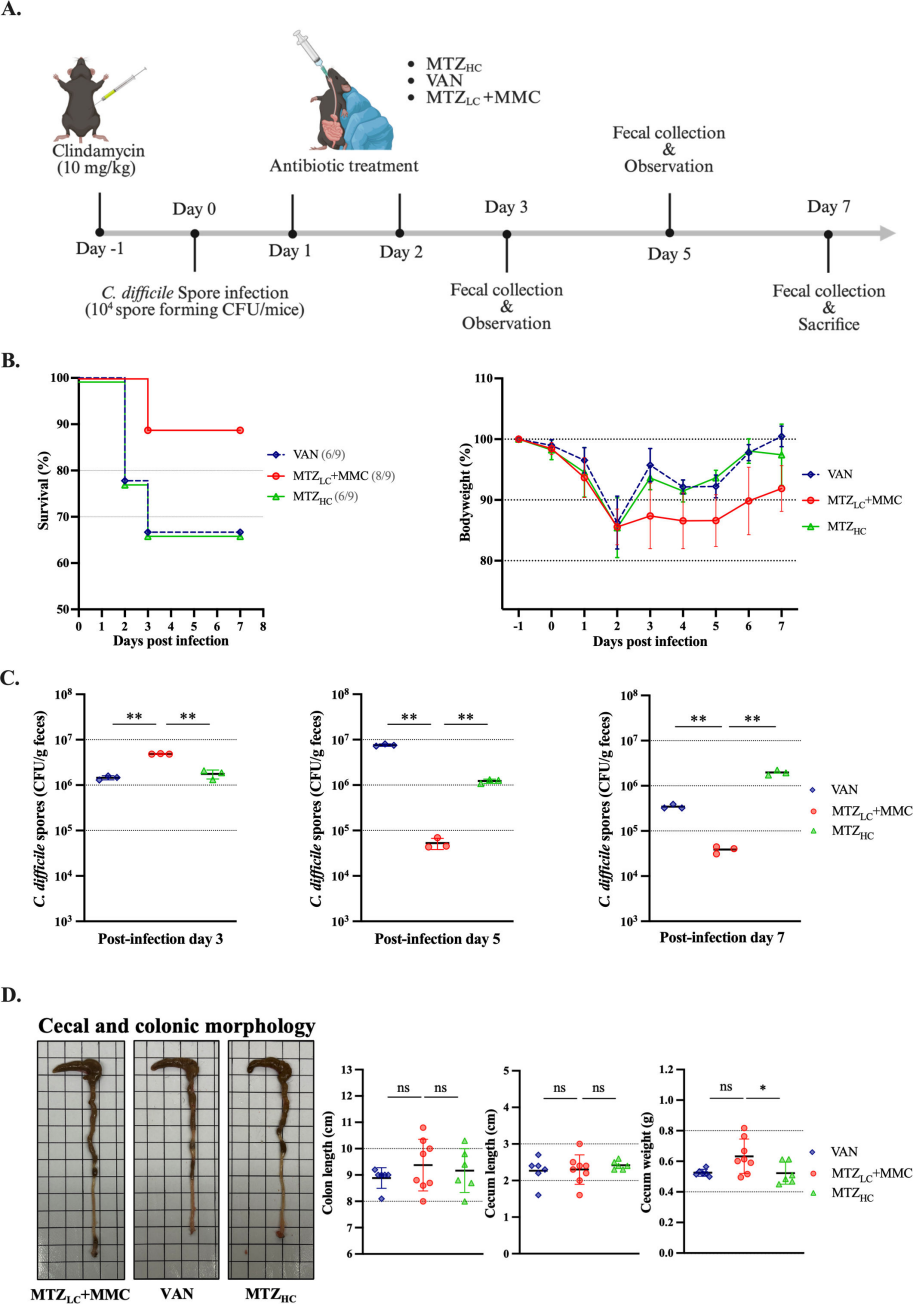
Comparison of MMC + MTZ_LC_ therapy with standard antibiotics in the treatment and prevention of CDI recurrence. (**A**) Diagram of the recurrent CDI mouse model (created with BioRender.com). Mice (*n* = 9 per group) were orally challenged with R20291 spores and treated with antibiotics for 2 days. (**B**) Survival rates and daily body weight changes post-infection. (**C**) Fecal spore-forming CFUs were measured on days 3, 5, and 7. (**D**) Representative images and quantification of cecal and colonic morphology. Each point represents one mouse. Statistical analysis was conducted using *t*-test and one-way ANOVA. *P ≤ 0.05, **P ≤ 0.01. ns, not significant.

Although VAN is considered more effective than MTZ in treating severe CDI (35, 36), its use is associated with gut microbiota disruption and increased recurrence risk (37, 38). We therefore compared MTZ_LC_ + MMC to VAN and MTZ_HC_ in the rCDI mouse model (Fig. 5A). The MTZ_LC_ + MMC group had the highest survival rate (89%) compared to the VAN and MTZ_HC_ groups (67% each) (Fig. 5B, left). Body weight decreased most in the VAN and MTZ_HC_ groups around days 3–4 post-infection, while this was less evident in the MTZ_LC_ + MMC group (Fig. 5B, right). All mice recovered to >90% of their initial weight by day 7. On day 3, the MTZ_LC_ + MMC group had higher spore-forming CFUs than the other groups (Fig. 5C, left). However, by days 5 and 7, the CFU counts in the MTZ_LC_ + MMC group were significantly lower than those in the VAN and MTZ_HC_ groups (Fig. 5C, middle and right). The VAN group showed a nearly 10-fold increase in fecal spores by day 5 post-infection, and the MTZ_HC_ group maintained high spore levels from day 3 to 7 post-infection. Histological analysis revealed no major differences in cecum and colon morphology, except for significantly greater cecal weight in the MTZ_LC_ + MMC group compared to the MTZ_HC_ group (Fig. 5D). Overall, the combination of low-dose MTZ with MMC yielded the best survival and spore reduction outcomes compared to VAN or MTZ alone.

### Combination therapy caused less host tissue damage and microbiota disruption

**Fig 6:**
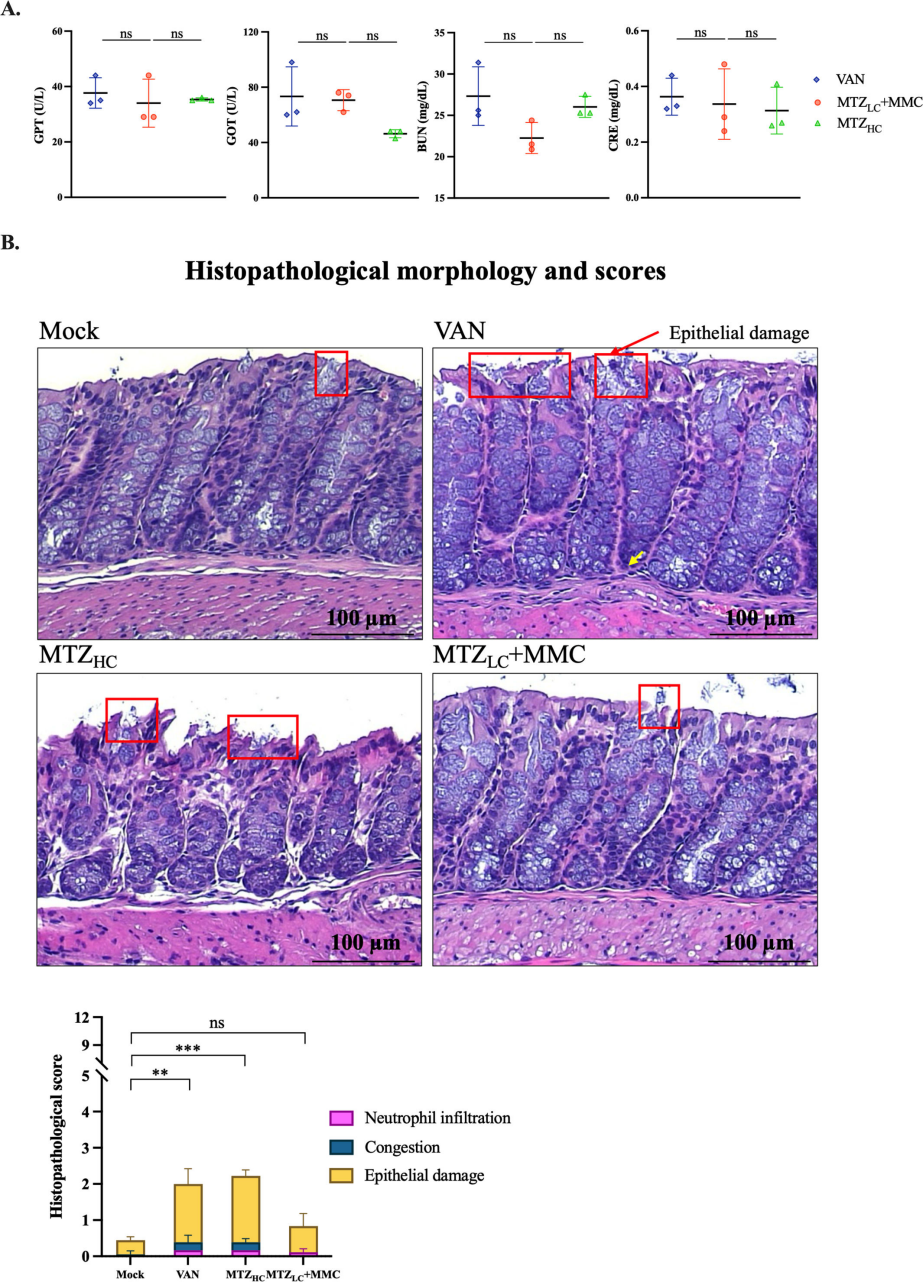
Evaluation of systemic and intestinal safety following antibiotic treatment. (**A**) Serum levels of GPT, GOT, BUN, and CRE were measured using a dry biochemical analyzer to assess liver and kidney functions. BUN, blood urea nitrogen; CRE, creatinine; GOT, glutamic oxaloacetic transaminase; GPT, glutamic pyruvic transaminase. (**B**) Yellow arrows indicate infiltrating neutrophils. Statistical analysis was performed using one-way ANOVA. **P ≤ 0.01, ***P ≤ 0.001. ns, not significant.

To ensure that MTZ_LC_ + MMC treatment did not induce greater systemic toxicity than other antibiotics, we collected serum samples on day 7 post-infection from VAN, MTZ_HC_, and MTZ_LC_ + MMC-treated mice. These samples were analyzed for GPT, GOT, BUN, and CRE. No significant differences were observed among the groups for any of these indicators (Fig. 6A). An additional experiment was conducted to evaluate intestinal damage. Mice were orally administered PBS (mock), low-dose MTZ + MMC, high-dose MTZ, or VAN for 2 days (Fig. S4A), and colonic tissues were histologically scored. All groups exhibited only mild epithelial damage; however, the MTZ_LC_ + MMC group had the lowest histopathological scores, comparable to those of the mock group (Fig. 6B). 16S rDNA sequencing revealed that the MTZ_LC_ + MMC group had the highest microbial similarity to the mock group, better than the VAN or MTZ_HC_ groups (Fig. S4B). Finally, intestinal length and weight measurements showed a significant increase in cecal weight in the VAN-treated group (Fig. S4C), consistent with prior findings of VAN-associated cecal enlargement (39). In summary, MTZ_LC_ + MMC caused less epithelial damage and systemic toxicity than other regimens and preserved gut microbiota composition more effectively.

## DISCUSSION

MTZ, a nitroimidazole antibiotic, has long been recognized for its potent activity against anaerobic bacterial infections (40). However, it is no longer recommended as a first-line treatment for CDI due to its comparatively lower efficacy and the potential for adverse pharmacological effects in humans, including nausea, vomiting, diarrhea, and, in some cases, neurotoxicity (41–43). Nevertheless, in Taiwan, MTZ is still clinically used to treat mild cases of CDI (44, 45). MTZ is more readily available and cost-effective compared to VAN and fidaxomicin for treating non-severe CDI (46). Moreover, our checkerboard assay demonstrated that combining MMC with MTZ synergistically inhibits *C. difficile* growth, while combining MMC and VAN only achieved additive effects. Notably, our *in vivo* results showed that the low-dose MMC + MTZ treatment achieved the highest survival rate and significantly reduced fecal spore counts compared to VAN or MTZ monotherapy. This suggests that combination therapy has the potential to be a novel therapeutic strategy. However, further trials are needed to determine the appropriate dosage and safety for humans in clinical usage.

CDI patients still rely on antibiotic treatments (2–4); however, these antibiotics disrupt gut microbiota and contribute to recurrence (47). This study found that MMC can synergistically cooperate with MTZ against *C. difficile*. Moreover, MMC enhanced the efficacy of MTZ in eliminating *C. difficile* within biofilms, despite biofilm formation protecting *C. difficile* against antibiotic attacks, which can result in recurrent infections (31–33, 48).

Our *in vivo* experiments showed that combining MTZ_LC_ with MMC increased mice survival rates after the spore challenge. Although survival was higher in the MTZ_LC_ + MMC group, the average body weight recovery appeared less pronounced. This observation may be due to survival bias in other groups, where animals with severe weight loss did not survive, thereby elevating the mean body weight of surviving individuals. Spore-forming CFUs in fecal samples, used to assess CDI recurrence, showed no significant difference between the R20291 and MTZ_LC_ + MMC groups after day 5 post-infection. Colonization resistance is critical for recovery and preventing rCDI, as a healthy microbiota plays an essential role in inhibiting *C. difficile* colonization and overgrowth (49–51). However, antibiotics disrupt microbiota diversity (52). Accordingly, we speculate that mice in both the R20291 and MTZ_LC_ + MMC groups may have experienced less microbiota disruption and thus recovered more effectively, helping prevent *C. difficile* colonization.

MTZ inhibits DNA synthesis (53), while MMC cross-links DNA to prevent bacterial replication (54). Our findings suggest that combining MTZ with MMC results in enhanced inhibition of bacterial DNA replication in *C. difficile*. Although MMC is known to exhibit toxicity across various organ systems, its toxicity profile depends on the route of administration and dosage (13, 55, 56). Clinically, MMC is primarily administered intravenously or intravesically (57–59). When given orally, it requires a dose 8–12 times higher than the intravenous route to achieve comparable activity (60). Previous studies have converted the standard clinical dose for humans (10 mg/m²) to an equivalent mouse dose, establishing an *in vivo* dosage of 2 mg/kg MMC (61). This translates to an oral dose of 16–24 mg/kg in mice, equivalent to 10 mg/m² in humans. In our study, we used an oral MMC dose of 5 mg/kg in mice, which is substantially lower than the clinical dosage, suggesting a potentially safer profile for oral use in future therapeutic applications.

In summary, our findings reveal a novel therapeutic strategy in which MMC enhances the efficacy of MTZ against CDI. Overexpression of NimB in some mutant strains has been reported to reduce the effectiveness of MTZ, contributing to antibiotic resistance (62). However, this resistance mechanism did not affect the efficacy of our MTZ-MMC combination treatment. Furthermore, the low dose of MTZ combined with MMC may help decrease the risk of rCDI by minimizing disruption to the gut microbiota (49, 51). Nevertheless, further studies are necessary to confirm the safety, optimal dosage, and potential side effects of the combined antibiotic therapy before advancing clinical application.

## SUPPLEMENTAL MATERIAL

Supplemental material aac.00515-25-s0001.pdf Fig. S1 to S4; Table S1.

ASM does not own the copyrights to Supplemental Material that may be linked to, or accessed through, an article. The authors have granted ASM a non-exclusive, world-wide license to publish the Supplemental Material files. Please contact the corresponding author directly for reuse.

## References

[1] Mounsey A, Lacy Smith K, Reddy VC, Nickolich S. 2020. Clostridioides difficile infection: update on management. Am Fam Physician 101:168–175. 32003951

[2] Kelly CR, Fischer M, Allegretti JR, LaPlante K, Stewart DB, Limketkai BN, Stollman NH. 2021. ACG clinical guidelines: prevention, diagnosis, and treatment of Clostridioides difficile infections. Am J Gastroenterol 116:1124–1147. doi:10.14309/ajg.0000000000001278 34003176

[3] McDonald LC, Gerding DN, Johnson S, Bakken JS, Carroll KC, Coffin SE, Dubberke ER, Garey KW, Gould CV, Kelly C, Loo V, Shaklee Sammons J, Sandora TJ, Wilcox MH. 2018. Clinical practice guidelines for Clostridium difficile infection in adults and children: 2017 update by the infectious diseases society of America (IDSA) and society for healthcare epidemiology of America (SHEA). Clin Infect Dis 66:e1–e48. doi:10.1093/cid/cix1085 29462280 PMC6018983

[4] Surawicz CM, Brandt LJ, Binion DG, Ananthakrishnan AN, Curry SR, Gilligan PH, McFarland LV, Mellow M, Zuckerbraun BS. 2013. Guidelines for diagnosis, treatment, and prevention of Clostridium difficile infections. Am J Gastroenterol 108:478–498. doi:10.1038/ajg.2013.4 23439232

[5] Chilton CH, Pickering DS, Freeman J. 2018. Microbiologic factors affecting Clostridium difficile recurrence. Clin Microbiol Infect 24:476–482. doi:10.1016/j.cmi.2017.11.017 29208562

[6] Feuerstadt P, Theriault N, Tillotson G. 2023. The burden of CDI in the United States: a multifactorial challenge. BMC Infect Dis 23:132. doi:10.1186/s12879-023-08096-0 36882700 PMC9990004

[7] Nelson WW, Scott TA, Boules M, Teigland C, Parente A, Unni S, Feuerstadt P. 2021. Health care resource utilization and costs of recurrent Clostridioides difficile infection in the elderly: a real-world claims analysis. J Manag Care Spec Pharm 27:828–838. doi:10.18553/jmcp.2021.20395 33703939 PMC10394752

[8] Song JH, Kim YS. 2019. Recurrent Clostridium difficile infection: risk factors, treatment, and prevention. Gut Liver 13:16–24. doi:10.5009/gnl18071 30400734 PMC6346998

[9] Feuerstadt P, Nelson WW, Drozd EM, Dreyfus J, Dahdal DN, Wong AC, Mohammadi I, Teigland C, Amin A. 2022. Mortality, health care use, and costs of Clostridioides difficile infections in older adults. J Am Med Dir Assoc 23:1721–1728. doi:10.1016/j.jamda.2022.01.075 35288083

[10] Polamreddy P, Gattu N. 2019. The drug repurposing landscape from 2012 to 2017: evolution, challenges, and possible solutions. Drug Discov Today 24:789–795. doi:10.1016/j.drudis.2018.11.022 30513339

[11] Cavalla D. 2019. Using human experience to identify drug repurposing opportunities: theory and practice. Br J Clin Pharmacol 85:680–689. doi:10.1111/bcp.13851 30648285 PMC6422651

[12] Gns HS, Gr S, Murahari M, Krishnamurthy M. 2019. An update on drug repurposing: re-written saga of the drug’s fate. Biomed Pharmacother 110:700–716. doi:10.1016/j.biopha.2018.11.127 30553197

[13] Verweij J, Pinedo HM. 1990. Mitomycin C: mechanism of action, usefulness and limitations. Anticancer Drugs 1:5–13. doi:10.1097/00001813-199010000-00002 2131038

[14] Metcalfe M, Wagenheim G, Xiao L, Papadopoulos J, Navai N, Davis JW, Karam JA, Kamat AM, Wood CG, Dinney CP, Matin SF. 2017. Induction and maintenance adjuvant mitomycin C topical therapy for upper tract urothelial carcinoma: tolerability and intermediate term outcomes. J Endourol 31:946–953. doi:10.1089/end.2016.0871 28731777 PMC5915259

[15] Read MD, Drake J, Hashemipour G, Powers BD, Mehta R, Sinnamon A, Pimiento JM, Dineen SP. 2024. Initial experience using laparoscopic HIPEC for gastric cancer with peritoneal metastasis: safety and outcomes. Ann Surg Oncol 31:3750–3757. doi:10.1245/s10434-024-15102-5 38430428

[16] Al-Humairi RMA, Hashim Mohammad T, Thanoon Ahmed S, Ad’hiah AH. 2023. Systemic interleukin-6 response after intravesical instillation of Bacillus Calmette-Guérin and mitomycin C in superficial bladder cancer. Arch Razi Inst 78:353–360. doi:10.22092/ARI.2022.358957.2344 37312727 PMC10258282

[17] Svedholm E, Bruce B, Parcell BJ, Coote PJ. 2024. Repurposing mitomycin C in combination with pentamidine or gentamicin to treat infections with multi-drug-resistant (MDR) Pseudomonas aeruginosa. Antibiotics (Basel) 13:177. doi:10.3390/antibiotics13020177 38391563 PMC10886254

[18] Pacios O, Fernández-García L, Bleriot I, Blasco L, González-Bardanca M, López M, Fernández-Cuenca F, Oteo J, Pascual Á, Martínez-Martínez L, Domingo-Calap P, Bou G, Tomás M, Study Group on Mechanisms of Action and Resistance to Antimicrobials (GEMARA) on behalf of the Spanish Society of Infectious Diseases and Clinical Microbiology (SEIMC). 2021. Enhanced antibacterial activity of repurposed mitomycin C and imipenem in combination with the lytic phage vB_KpnM-VAC13 against clinical isolates of Klebsiella pneumoniae. Antimicrob Agents Chemother 65:e0090021. doi:10.1128/AAC.00900-21 34228538 PMC8370222

[19] Pal R, Seleem MN. 2020. Screening of natural products and approved oncology drug libraries for activity against Clostridioides difficile. Sci Rep 10:5966. doi:10.1038/s41598-020-63029-0 32249833 PMC7136261

[20] Domalaon R, Ammeter D, Brizuela M, Gorityala BK, Zhanel GG, Schweizer F. 2019. Repurposed antimicrobial combination therapy: tobramycin-ciprofloxacin hybrid augments activity of the anticancer drug mitomycin C against multidrug-resistant gram-negative bacteria. Front Microbiol 10:1556. doi:10.3389/fmicb.2019.01556 31354660 PMC6636613

[21] Hu Y, Liu Y, Coates A. 2019. Azidothymidine produces synergistic activity in combination with colistin against antibiotic-resistant Enterobacteriaceae. Antimicrob Agents Chemother 63:e01630-18. doi:10.1128/AAC.01630-18 30373798 PMC6325217

[22] Odds FC. 2003. Synergy, antagonism, and what the chequerboard puts between them. J Antimicrob Chemother 52:1. doi:10.1093/jac/dkg301 12805255

[23] Nachnani S, Scuteri A, Newman MG, Avanessian AB, Lomeli SL. 1992. E-test: a new technique for antimicrobial susceptibility testing for periodontal microorganisms. J Periodontol 63:576–583. doi:10.1902/jop.1992.63.7.576 1324301

[24] Lambert RJ, Pearson J. 2000. Susceptibility testing: accurate and reproducible minimum inhibitory concentration (MIC) and non-inhibitory concentration (NIC) values. J Appl Microbiol 88:784–790. doi:10.1046/j.1365-2672.2000.01017.x 10792538

[25] Gong J-J, Huang I-H, Su MS-W, Xie S-X, Liu W-Y, Huang C-R, Hung Y, Wu S-R, Tsai P, Ko W, Chen J-W. 2024. Phage transcriptional regulator X (PtrX)-mediated augmentation of toxin production and virulence in Clostridioides difficile strain R20291. Microbiol Res 280:127576. doi:10.1016/j.micres.2023.127576 38183754

[26] Permpoonpattana P, Tolls EH, Nadem R, Tan S, Brisson A, Cutting SM. 2011. Surface layers of Clostridium difficile endospores. J Bacteriol 193:6461–6470. doi:10.1128/JB.05182-11 21949071 PMC3232898

[27] Bublitz A, Brauer M, Wagner S, Hofer W, Müsken M, Deschner F, Lesker TR, Neumann-Schaal M, Paul LS, Nübel U, Bartel J, Kany AM, Zühlke D, Bernecker S, Jansen R, Sievers S, Riedel K, Herrmann J, Müller R, Fuchs TM, Strowig T. 2023. The natural product chlorotonil A preserves colonization resistance and prevents relapsing Clostridioides difficile infection. Cell Host Microbe 31:734–750. doi:10.1016/j.chom.2023.04.003 37098342

[28] Shelby RD, Tengberg N, Conces M, Olson JK, Navarro JB, Bailey MT, Goodman SD, Besner GE. 2020. Development of a standardized scoring system to assess a murine model of Clostridium difficile colitis. J Invest Surg 33:887–895. doi:10.1080/08941939.2019.1571129 30892111

[29] Tsai BY, Lai YH, Chiu CW, Hsu CY, Chen YH, Chen YL, Tsai PJ, Hung YP, Ko WC. 2022. Effect of doxycycline in decreasing the severity of Clostridioides difficile infection in mice. Antibiotics (Basel) 11:116. doi:10.3390/antibiotics11010116 35052993 PMC8772929

[30] Hargreaves KR, Colvin HV, Patel KV, Clokie JJP, Clokie MRJ. 2013. Genetically diverse Clostridium difficile strains harboring abundant prophages in an estuarine environment. Appl Environ Microbiol 79:6236–6243. doi:10.1128/AEM.01849-13 23913427 PMC3811203

[31] Soavelomandroso AP, Gaudin F, Hoys S, Nicolas V, Vedantam G, Janoir C, Bouttier S. 2017. Biofilm structures in a mono-associated mouse model of Clostridium difficile infection. Front Microbiol 8:2086. doi:10.3389/fmicb.2017.02086 29118745 PMC5661025

[32] Morais MLGDS, Santos MGC, Costa CL, Martins CS, Leitão RF de C, de Melo Pacífico D, Quesada-Gómez C, Castelo Branco D, Ferreira E de O, Brito GA de C. 2022. Comparative biofilm-forming ability between Clostridioides difficile strains isolated in Latin America and the epidemic NAP1/027 strain. Front Cell Infect Microbiol 12:1033698. doi:10.3389/fcimb.2022.1033698 36619751 PMC9815708

[33] Ðapa T, Leuzzi R, Ng YK, Baban ST, Adamo R, Kuehne SA, Scarselli M, Minton NP, Serruto D, Unnikrishnan M. 2013. Multiple factors modulate biofilm formation by the anaerobic pathogen Clostridium difficile. J Bacteriol 195:545–555. doi:10.1128/JB.01980-12 23175653 PMC3554014

[34] Huang J, Kelly CP, Bakirtzi K, Villafuerte Gálvez JA, Lyras D, Mileto SJ, Larcombe S, Xu H, Yang X, Shields KS, Zhu W, Zhang Y, Goldsmith JD, Patel IJ, Hansen J, Huang M, Yla-Herttuala S, Moss AC, Paredes-Sabja D, Pothoulakis C, Shah YM, Wang J, Chen X. 2019. Clostridium difficile toxins induce VEGF-A and vascular permeability to promote disease pathogenesis. Nat Microbiol 4:269–279. doi:10.1038/s41564-018-0300-x 30510170 PMC6559218

[35] Di X, Bai N, Zhang X, Liu B, Ni W, Wang J, Wang K, Liang B, Liu Y, Wang R. 2015. A meta-analysis of metronidazole and vancomycin for the treatment of Clostridium difficile infection, stratified by disease severity. Braz J Infect Dis 19:339–349. doi:10.1016/j.bjid.2015.03.006 26001980 PMC9427463

[36] Erikstrup LT, Aarup M, Hagemann-Madsen R, Dagnaes-Hansen F, Kristensen B, Olsen KEP, Fuursted K. 2015. Treatment of Clostridium difficile infection in mice with vancomycin alone is as effective as treatment with vancomycin and metronidazole in combination. BMJ Open Gastroenterol 2:e000038. doi:10.1136/bmjgast-2015-000038 PMC4641438 26568840

[37] Lewis BB, Buffie CG, Carter RA, Leiner I, Toussaint NC, Miller LC, Gobourne A, Ling L, Pamer EG. 2015. Loss of microbiota-mediated colonization resistance to Clostridium difficile infection with oral vancomycin compared with metronidazole. J Infect Dis 212:1656–1665. doi:10.1093/infdis/jiv256 25920320 PMC4621244

[38] Warren CA, van Opstal EJ, Riggins MS, Li Y, Moore JH, Kolling GL, Guerrant RL, Hoffman PS. 2013. Vancomycin treatment’s association with delayed intestinal tissue injury, clostridial overgrowth, and recurrence of Clostridium difficile infection in mice. Antimicrob Agents Chemother 57:689–696. doi:10.1128/AAC.00877-12 23147742 PMC3553708

[39] Xu X, Fukui H, Ran Y, Tomita T, Oshima T, Watari J, Miwa H. 2019. Alteration of GLP-1/GPR43 expression and gastrointestinal motility in dysbiotic mice treated with vancomycin. Sci Rep 9:4381. doi:10.1038/s41598-019-40978-9 30867532 PMC6416360

[40] Leitsch D. 2019. A review on metronidazole: an old warhorse in antimicrobial chemotherapy. Parasitology 146:1167–1178. doi:10.1017/S0031182017002025 29166971

[41] AlDhaleei W, AlMarzooqi A, Gaber N. 2018. Reversible metronidazole-induced neurotoxicity after 10 weeks of therapy. BMJ Case Rep 2018:bcr2017223463. doi:10.1136/bcr-2017-223463 PMC5911128 29678819

[42] Quickfall D, Daneman N, Dmytriw AA, Juurlink DN. 2021. Metronidazole-induced neurotoxicity. CMAJ 193:E1630. doi:10.1503/cmaj.201671 34697097 PMC8562982

[43] Sørensen CG, Karlsson WK, Amin FM, Lindelof M. 2020. Metronidazole-induced encephalopathy: a systematic review. J Neurol 267:1–13. doi:10.1007/s00415-018-9147-6 30536109

[44] Lee HY, Hsiao HL, Chia CY, Cheng CW, Tsai TC, Deng ST, Chen CL, Chiu CH. 2019. Risk factors and outcomes of Clostridium difficile infection in hospitalized patients. Biomed J 42:99–106. doi:10.1016/j.bj.2018.12.002 31130254 PMC6541878

[45] McDonald LC, Gerding DN, Johnson S, Bakken JS, Carroll KC, Coffin SE, Dubberke ER, Garey KW, Gould CV, Kelly C, Loo V, Shaklee Sammons J, Sandora TJ, Wilcox MH. 2018. Clinical Practice Guidelines for Clostridium difficile Infection in Adults and Children: 2017 Update by the Infectious Diseases Society of America (IDSA) and Society for Healthcare Epidemiology of America (SHEA). Clin Infect Dis 66:987–994. doi:10.1093/cid/ciy149 29562266

[46] Varier RU, Biltaji E, Smith KJ, Roberts MS, Jensen MK, LaFleur J, Nelson RE. 2014. Cost-effectiveness analysis of treatment strategies for initial Clostridium difficile infection. Clin Microbiol Infect 20:1343–1351. doi:10.1111/1469-0691.12805 25366338

[47] Berkell M, Mysara M, Xavier BB, van Werkhoven CH, Monsieurs P, Lammens C, Ducher A, Vehreschild MJGT, Goossens H, de Gunzburg J, Bonten MJM, Malhotra-Kumar S, ANTICIPATE study group. 2021. Microbiota-based markers predictive of development of Clostridioides difficile infection. Nat Commun 12:2241. doi:10.1038/s41467-021-22302-0 33854066 PMC8047037

[48] Dawson LF, Peltier J, Hall CL, Harrison MA, Derakhshan M, Shaw HA, Fairweather NF, Wren BW. 2021. Extracellular DNA, cell surface proteins and c-di-GMP promote biofilm formation in Clostridioides difficile*.* Sci Rep 11:3244. doi:10.1038/s41598-020-78437-5 33547340 PMC7865049

[49] Theriot CM, Koenigsknecht MJ, Carlson PE Jr, Hatton GE, Nelson AM, Li B, Huffnagle GB, Z Li J, Young VB. 2014. Antibiotic-induced shifts in the mouse gut microbiome and metabolome increase susceptibility to Clostridium difficile infection. Nat Commun 5:3114. doi:10.1038/ncomms4114 24445449 PMC3950275

[50] Britton RA, Young VB. 2014. Role of the intestinal microbiota in resistance to colonization by Clostridium difficile. Gastroenterology 146:1547–1553. doi:10.1053/j.gastro.2014.01.059 24503131 PMC3995857

[51] Theriot CM, Young VB. 2015. Interactions between the gastrointestinal microbiome and Clostridium difficile. Annu Rev Microbiol 69:445–461. doi:10.1146/annurev-micro-091014-104115 26488281 PMC4892173

[52] Binyamin D, Nitzan O, Azrad M, Hamo Z, Koren O, Peretz A. 2021. The microbial diversity following antibiotic treatment of Clostridioides difficile infection. BMC Gastroenterol 21:166. doi:10.1186/s12876-021-01754-0 33849457 PMC8045228

[53] Edwards DI. 1979. Mechanism of antimicrobial action of metronidazole. J Antimicrob Chemother 5:499–502. doi:10.1093/jac/5.5.499 387703

[54] Tomasz M, Palom Y. 1997. The mitomycin bioreductive antitumor agents: cross-linking and alkylation of DNA as the molecular basis of their activity. Pharmacol Ther 76:73–87. doi:10.1016/s0163-7258(97)00088-0 9535170

[55] Nissenkorn I, Herrod H, Soloway MS. 1981. Side effects associated with intravesical mitomycin. J Urol 126:596–597. doi:10.1016/s0022-5347(17)54642-x 6457917

[56] Wu KY, Hong SJ, Huang HT, Lin CP, Chen CW. 1999. Toxic effects of mitomycin-C on cultured corneal keratocytes and endothelial cells. J Ocul Pharmacol Ther 15:401–411. doi:10.1089/jop.1999.15.401 10530701

[57] Shen Z, Shen T, Wientjes MG, O’Donnell MA, Au JL-S. 2008. Intravesical treatments of bladder cancer: review. Pharm Res 25:1500–1510. doi:10.1007/s11095-008-9566-7 18369709 PMC2440939

[58] Hortobagyi GN. 1985. Mitomycin-C in breast cancer. Semin Oncol 12:65–70. 3936183

[59] Sinawe H, Casadesus D. 2025. Mitomycin. StatPearls, Treasure Island, FL. https://www.ncbi.nlm.nih.gov/books/NBK562249/.

[60] Crooke ST, Bradner WT. 1976. Mitomycin C: a review. Cancer Treat Rev 3:121–139. doi:10.1016/s0305-7372(76)80019-9 786455

[61] Chen F-S, Chi C-W, Shieh H-R, Lin C-P, Ko C-C, Chung Y-C, Lai J-Y, Tai H-C, Chen Y-J. 2019. Mitomycin C modulates tumor microenvironment and enhances radiosensitivity in rectal cancer. Ther Radiol Oncol 3:29–29. doi:10.21037/tro.2019.07.05

[62] Olaitan AO, Dureja C, Youngblom MA, Topf MA, Shen WJ, Gonzales-Luna AJ, Deshpande A, Hevener KE, Freeman J, Wilcox MH, Palmer KL, Garey KW, Pepperell CS, Hurdle JG. 2023. Decoding a cryptic mechanism of metronidazole resistance among globally disseminated fluoroquinolone-resistant Clostridioides difficile. Nat Commun 14:4130. doi:10.1038/s41467-023-39429-x 37438331 PMC10338468

