# Supplementary material for: Combination of mitomycin C and low-dose metronidazole synergistically against Clostridioides difficile infection and recurrence prevention
Source: Antimicrob Agents Chemother. 2025 Jun 17;69(8):e00515-25. doi: 10.1128/aac.00515-25 (PMC12326999; doi:10.1128/aac.00515-25)
Supplement: Supplemental material — Fig. S1 to S4; Table S1. [file aac.00515-25-s0001.pdf]

Supplementary Information

Figure S1. In vitro evaluation of MMC assisted antibiotics against *C. difficile* growth. (A) The checkerboard assay was performed to determine the growth of RT027 and RT078 *C. difficile* clinical strains after being treated with different antibiotic concentrations. (B) VAN+MMC efficacy inhibited RT078 clinical strains was evaluated using the checkerboard assay.

Figure S2. No viable bacteria detected in extracellular biofilm after treatment with MTZ and MMC.

Figure S3. Preparation of CDI recurrence model and observation of mouse health status. (A) Nycodenz-purified R20291 spores were stained with malachite green. Green: R20291 spores. Pink: R20291 vegetative cell debris. (B) Spores were germinated on 0.1% taurocholic acid BHIS agar to calculate the CFU counts. (C) Daily monitoring of mouse clinical sickness score (CSS) from the 3 to 7 days post infection.

Figure S4. Animal model for evaluating the side effects caused by antibiotic treatments. (**A**) Schematic diagram of the antibiotic-treated mouse model (Created with BioRender.com). (**B**) Hierarchical clustering heatmap of mice gut microbial

composition. Low abundance taxa are shown as blue and high abundance as yellow. The top 40 most abundant taxa (combined across samples) are shown. Taxa must have at least one sample with a relative abundance of >=1% to be shown. Rows and columns are hierarchically clustered with samples more similar to each other clustered closer together. Dendogram branch lengths indicate distance between clusters. (**C**) The quantification of cecal and colonic tissues. All intestinal tissues were harvested when the mice were sacrificed on day 4 post-antibiotic treatment. Each point represents the data of one mouse. Statistical analysis was performed using GraphPad Prism 10.0, and significance was determined by the one-way analysis of variance (*≤ 0.05; ****$p \leq 0.0001$; ns, not significant).

Supplemental figures

Figure S1A.

**RT027 R20291**													
MTZ (µg/mL)													
6	11	10	10	11	11	11	11	11	11	11	11	10	
3	12	12	11	11	11	11	11	11	11	11	11	10	
1.5	64	70	65	67	54	61	67	12	12	12	13	14	
0.75	91	99	98	97	97	97	87	68	11	12	13	14	
0.375	91	91	94	93	91	98	77	53	11	12	13	15	
0.1875	111	101	103	100	100	100	87	86	24	12	13	15	
0.09375	102	108	107	105	105	102	95	98	83	12	14	15	
0.046875	Drug Free	103	116	105	104	102	91	91	95	11	12	14	
	0.004	0.078	0.016	0.031	0.063	0.125	0.25	0.5	1	2	4	8	MMC (µg/mL)
**RT027 CMMC-41**													
MTZ (µg/mL)													
6	17	18	18	17	17	18	18	18	17	18	18	19	
3	17	17	17	17	17	17	17	17	17	18	18	19	
1.5	72	74	73	71	63	65	55	21	17	17	18	19	
0.75	82	79	79	82	83	84	83	89	86	17	18	19	
0.375	87	85	85	85	87	87	86	92	84	75	18	19	
0.1875	89	87	88	88	87	87	88	90	83	92	18	19	
0.09375	94	91	93	92	93	93	93	91	89	93	24	19	
0.046875	100	102	102	99	101	100	101	99	94	97	75	19	
	0.004	0.078	0.016	0.031	0.063	0.125	0.25	0.5	1	2	4	8	MMC (µg/mL)
**RT027 CMMC-47**													
MTZ (µg/mL)													
6	19	20	20	19	20	19	20	19	19	20	20	21	
3	19	19	19	19	19	19	19	19	19	19	20	21	
1.5	33	34	48	43	41	42	36	20	19	19	20	21	
0.75	88	87	87	92	88	87	83	82	23	20	20	21	
0.375	89	89	89	89	89	90	90	88	76	19	20	21	
0.1875	94	89	91	92	91	91	90	88	89	29	20	21	
0.09375	92	93	91	92	93	92	93	90	91	69	21	21	
0.046875	100	98	100	99	99	100	98	97	94	95	21	22	
	0.004	0.078	0.016	0.031	0.063	0.125	0.25	0.5	1	2	4	8	MMC (µg/mL)

**RT027 NCKUH-118**													
MTZ (µg/mL)													
6	18	19	18	18	18	18	18	20	18	19	18	18	
3	18	18	18	18	18	18	18	18	18	18	19	20	
1.5	86	96	89	87	87	87	87	78	22	18	19	20	
0.75	94	88	89	90	92	92	94	93	79	26	19	19	
0.375	94	92	89	91	91	92	94	91	80	92	20	20	
0.1875	97	90	92	94	95	95	96	94	94	95	26	20	
0.09375	97	96	95	94	97	97	99	97	96	97	46	19	
0.046875	100	99	100	100	103	108	107	106	104	103	81	20	
	0.004	0.078	0.016	0.031	0.063	0.125	0.25	0.5	1	2	4	8	MMC (µg/mL)

**RT027 NTU-50**													
MTZ (µg/mL)													
6	22	22	22	21	21	21	22	22	22	23	24	25	
3	21	21	21	21	21	21	21	21	21	22	23	25	
1.5	21	21	22	21	21	21	21	21	21	21	22	24	
0.75	95	85	82	84	83	89	92	93	105	21	23	23	
0.375	86	84	70	72	71	72	72	75	97	27	23	24	
0.1875	87	83	81	84	81	83	70	82	81	71	23	25	
0.09375	89	88	86	84	83	85	78	76	81	98	23	23	
0.046875	100	99	95	91	94	96	91	89	79	83	70	24	
	0.004	0.078	0.016	0.031	0.063	0.125	0.25	0.5	1	2	4	8	MMC (µg/mL)

**RT027 CMMC-93**													
MTZ (µg/mL)													
6	23	23	24	23	23	23	23	23	23	23	24	25	
3	22	22	22	22	22	22	22	22	22	22	23	25	
1.5	22	22	23	22	22	22	22	21	22	22	22	25	
0.75	43	70	95	84	81	83	41	21	22	22	23	24	
0.375	91	83	86	86	86	88	83	98	21	22	22	24	
0.1875	90	86	87	87	78	72	68	87	98	22	22	24	
0.09375	91	89	87	86	82	82	79	83	90	24	22	24	
0.046875	100	97	95	94	80	92	92	77	81	101	25	24	
	0.004	0.078	0.016	0.031	0.063	0.125	0.25	0.5	1	2	4	8	MMC (µg/mL)

**RT078_83**													
MTZ (µg/mL)													
6	13	14	14	13	13	13	14	14	14	14	14	14	
3	13	14	14	14	13	13	13	14	14	14	15	15	
1.5	13	13	14	14	13	13	14	13	14	14	14	14	
0.75	13	14	14	14	13	14	14	13	13	13	14	14	
0.375	85	88	13	13	13	13	13	14	13	14	14	14	
0.1875	102	106	96	94	89	97	16	14	14	14	14	14	
0.09375	89	100	101	91	94	97	93	84	14	14	14	14	
0.046875	100	91	92	92	87	96	102	109	55	13	14	14	
	0.004	0.078	0.016	0.031	0.063	0.125	0.25	0.5	1	2	4	8	MMC (µg/mL)
**RT078_84**													
MTZ (µg/mL)													
6	11	12	12	12	11	11	12	12	12	12	13	13	
3	11	12	12	12	12	12	12	12	12	12	12	12	
1.5	12	11	11	12	12	12	12	12	12	12	12	12	
0.75	11	12	11	11	12	12	12	12	12	12	12	12	
0.375	55	50	12	12	12	12	12	12	12	12	12	12	
0.1875	83	85	86	92	100	36	63	19	13	13	13	13	
0.09375	88	86	82	83	92	93	88	86	12	12	13	13	
0.046875	100	86	85	86	89	83	90	102	11	12	12	12	
	0.004	0.078	0.016	0.031	0.063	0.125	0.25	0.5	1	2	4	8	MMC (µg/mL)
**RT078_85**													
MTZ (µg/mL)													
6	13	14	14	13	13	13	13	13	13	13	14	13	
3	13	13	14	14	13	13	13	13	13	14	14	13	
1.5	13	13	13	14	13	13	13	13	14	14	14	13	
0.75	13	14	13	13	13	13	13	13	13	14	14	13	
0.375	13	13	13	13	13	14	14	13	13	14	14	14	
0.1875	80	52	44	24	13	13	14	13	14	14	14	14	
0.09375	96	93	91	93	13	13	13	13	14	14	14	13	
0.046875	100	96	98	101	97	65	13	13	13	13	13	13	
	0.004	0.078	0.016	0.031	0.063	0.125	0.25	0.5	1	2	4	8	MMC (µg/mL)

**RT078_86**													
MTZ (µg/mL)													
6	13	14	13	13	13	13	14	13	14	14	14	14	
3	13	13	13	13	13	12	14	13	14	14	14	14	
1.5	13	13	13	13	13	13	14	13	13	13	14	14	
0.75	13	13	13	13	13	13	13	13	13	13	14	14	
0.375	95	89	98	99	93	42	14	14	14	14	15	15	
0.1875	84	83	84	83	88	93	96	76	15	15	15	15	
0.09375	85	85	80	86	88	90	98	91	15	14	15	15	
0.046875	100	98	94	95	89	88	95	93	98	14	14	14	
	0.004	0.078	0.016	0.031	0.063	0.125	0.25	0.5	1	2	4	8	MMC (µg/mL)
**RT078_87**													
MTZ (µg/mL)													
6	13	13	13	13	13	13	13	13	13	13	14	14	
3	13	13	13	13	13	13	13	13	13	13	13	13	
1.5	13	13	13	14	13	12	13	12	13	13	13	13	
0.75	13	13	13	12	13	13	13	13	13	13	13	13	
0.375	98	99	95	99	99	97	18	14	13	14	14	14	
0.1875	96	106	97	104	104	105	105	95	14	14	15	15	
0.09375	99	103	101	108	99	101	102	102	95	15	15	15	
0.046875	100	101	97	107	105	102	103	102	105	13	13	13	
	0.004	0.078	0.016	0.031	0.063	0.125	0.25	0.5	1	2	4	8	MMC (µg/mL)

Figure S1B.

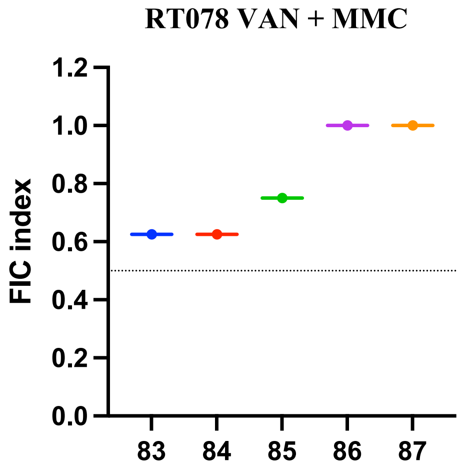

Figure S2.

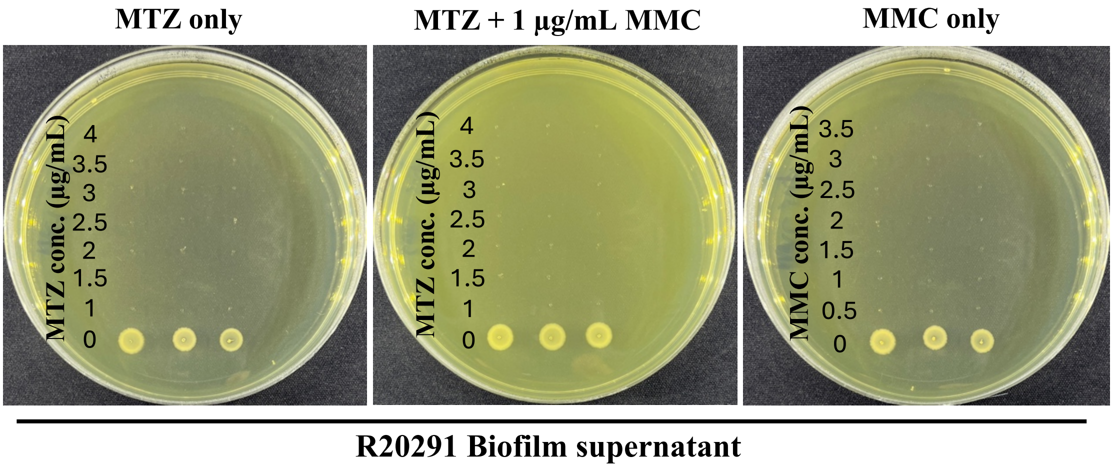

Figure S3A.

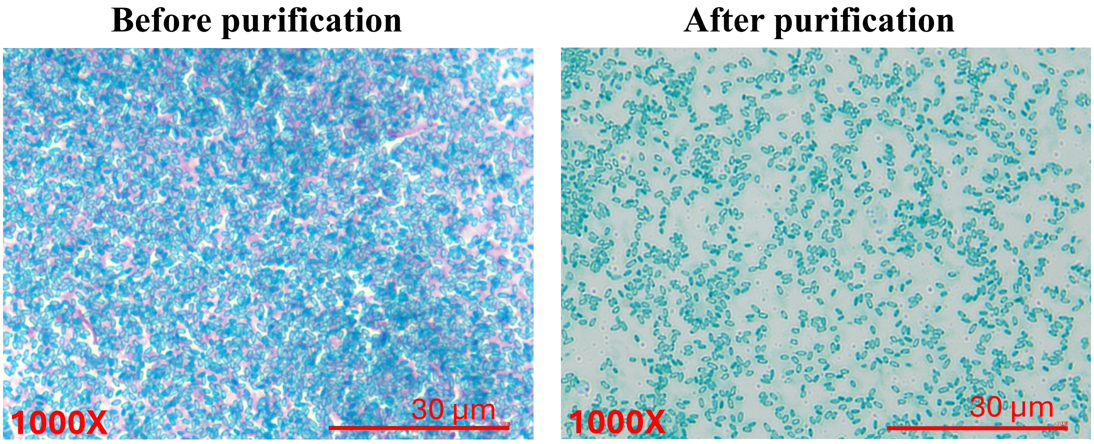

Figure S3B.

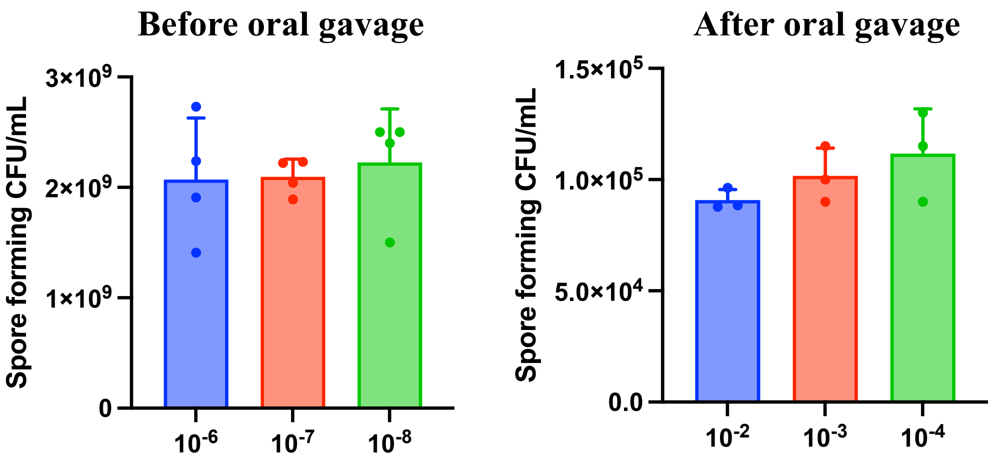
Before oral gavage
After oral gavage
Spore forming CFU/mL
3×10^9
2×10^9
1×10^9
0
10^-6
10^-7
10^-8
Spore forming CFU/mL
1.5×10^5
1.0×10^5
5.0×10^4
0.0
10^-2
10^-3
10^-4

Figure S3C.

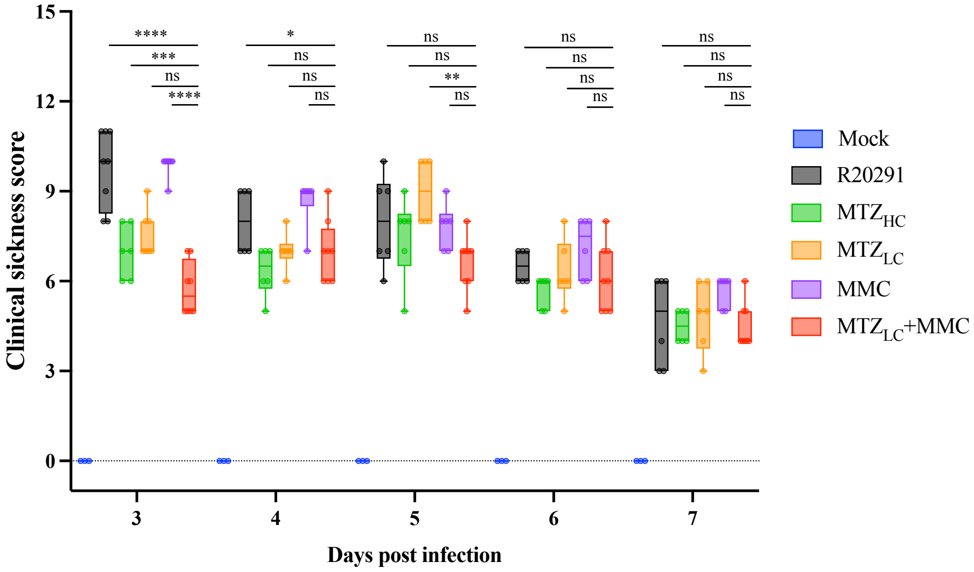
Clinical sickness score
15
12
9
6
3
0
****
***
ns
****
*
ns
ns
ns
ns
ns
**
ns
ns
ns
ns
ns
ns
ns
ns
ns
3
4
5
6
7
Days post infection
Mock
R20291
MTZ_HC
MTZ_LC
MMC
MTZ_LC+MMC

Figure S4A.

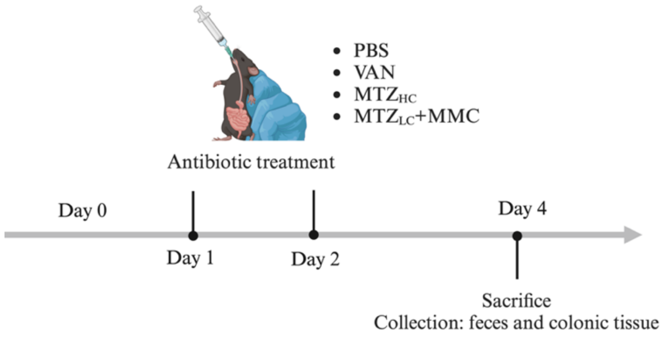

Figure S4B.

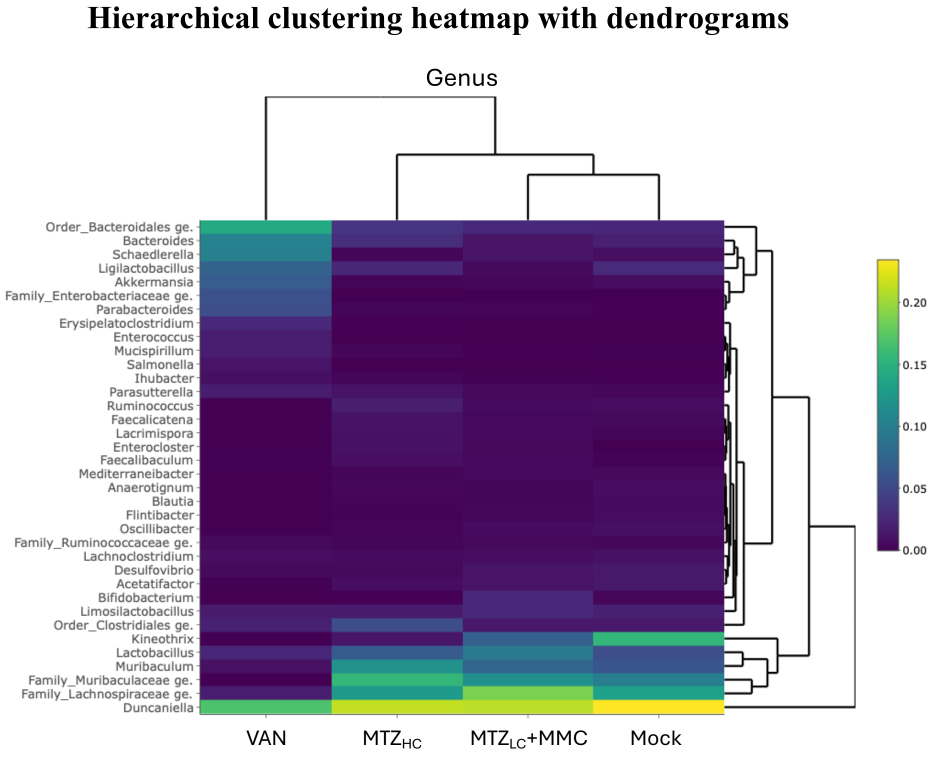

Figure S4C.

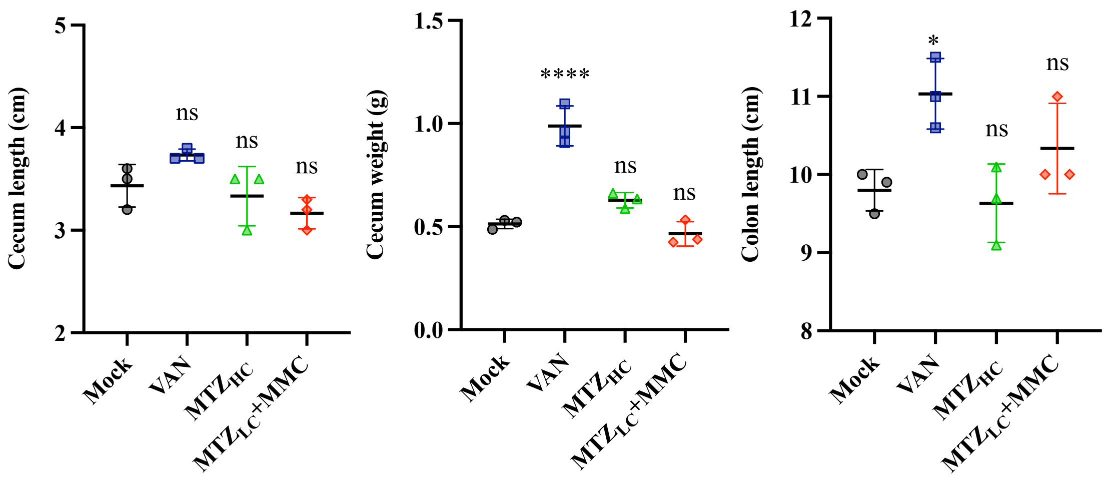

Supplemental tables

Table S1. Bacterial strains used in this study

Bacterial strain	Description	Source
RT027 *C. difficile* strains		
R20291	$A^+B^+CDT^+$, RT027	Dr. I-Hsiu Huang
CMMC-41	$A^+B^+CDT^+$, RT027	Dr. Yuan-Pin Hung
CMMC-47	$A^+B^+CDT^+$, RT027	Dr. Yuan-Pin Hung
NCKUH-93	$A^+B^+CDT^+$, RT027	Dr. Yuan-Pin Hung
NCKUH-118	$A^+B^+CDT^+$, RT027	Dr. Yuan-Pin Hung
NTU-50	$A^+B^+CDT^+$, RT027	Dr. Yuan-Pin Hung
RT078 *C. difficile* clinical strains		
RT078_83	$A^+B^+CDT^+$, RT078	Dr. Yuan-Pin Hung
RT078_84	$A^+B^+CDT^+$, RT078	Dr. Yuan-Pin Hung
RT078_85	$A^+B^+CDT^+$, RT078	Dr. Yuan-Pin Hung
RT078_86	$A^+B^+CDT^+$, RT078	Dr. Yuan-Pin Hung
RT078_87	$A^+B^+CDT^+$, RT078	Dr. Yuan-Pin Hung
